# Infiltrative Endometriosis Clinically Mimicking Ovarian Malignancy: Diagnostic Utility of Intraoperative Frozen Section

**DOI:** 10.7759/cureus.99229

**Published:** 2025-12-14

**Authors:** Aparna Gupta, Reena Tomar, Robin Yadav, Deepika Rani, Asmita Rathore

**Affiliations:** 1 Obstetrics and Gynaecology, Maulana Azad Medical College, New Delhi, IND; 2 Pathology, Maulana Azad Medical College, New Delhi, IND

**Keywords:** frozen section diagnosis, high ca 125, infiltrative endometriosis, ovarian cancer mimic, serum tumour markers

## Abstract

Endometriosis is a benign gynecological condition that commonly affects women of reproductive age. It is characterized by the presence of ectopic endometrial tissue, predominantly within the pelvic cavity. Ovarian endometriosis usually presents as an endometrioma with typical clinical and radiological features. However, atypical presentations such as varied symptoms, elevated tumor markers, and inconclusive imaging often complicate the differentiation between benign and malignant ovarian masses. This poses significant diagnostic challenges, which can be addressed with the use of intraoperative frozen sections. We hereby report a case of infiltrative endometriosis that presented with clinical features, radiological findings, and elevated serum tumor markers suggestive of ovarian malignancy.

## Introduction

Endometriosis is a common gynecological condition characterized by the presence of functional endometrial tissue outside the uterus. It affects 10-15% of reproductive-age women - approximately 176 million worldwide [1]. The exact etiopathogenesis remains unknown, although several theories have been proposed, including coelomic metaplasia, hormonal induction, and Sampson’s theory of retrograde menstruation.

Ectopic endometrial tissue is usually located within the pelvic cavity, with the ovary being the most commonly affected site. Other frequent locations include the posterior broad ligament, cul-de-sac, and uterosacral ligaments. Extrapelvic sites such as the gastrointestinal tract, abdominal wall, pleura, pericardium, and central nervous system may also be involved, although rarely.

Ovarian endometriosis typically presents with chronic pelvic pain, dysmenorrhea, dyspareunia, and infertility. On clinical examination, it may manifest as an ovarian mass referred to as an endometrioma. Ultrasound imaging characteristically shows a “ground glass” appearance due to debris from cyclical proliferation and shedding of endometrial tissue. Serological evaluation may reveal mildly elevated CA-125 levels, which correlate with the stage of the disease [2]. However, the gold standard for diagnosis remains direct surgical visualization followed by histopathological examination, which demonstrates endometrial glands and stroma [3].

The disease can have variable presentations, posing significant diagnostic challenges and often leading to delays in diagnosis. Ovarian endometriomas may appear atypical on imaging, exhibiting features such as internal echoes or septations that make them difficult to distinguish from malignancy [4]. In such cases, intraoperative frozen section analysis can be valuable in differentiating benign from malignant lesions, enabling more conservative, tissue-preserving surgical approaches.

This report discusses a case of infiltrative endometriosis that clinically, radiologically, and biochemically mimicked ovarian malignancy.

## Case presentation

A 40-year-old woman, para 2 living 2, with no abortions (P2L2A0), presented to the Gynecology Outpatient Department with chief complaints of pelvic pain and dysmenorrhea for the past six months. She had no history of systemic diseases such as diabetes, hypertension, thyroid disorders, or coronary artery disease. On clinical examination, a mass was palpated in the right lower quadrant of the abdomen.

Ultrasound imaging revealed a hypoechoic lesion postero-inferior to the uterus, measuring 2 × 1.7 cm, suggestive of a fibroid. Additionally, a large multiloculated solid-cystic lesion was noted in the right ovary, measuring 14 × 13 × 8.4 cm, with a solid component measuring 5.2 × 4.2 × 3.6 cm. Contrast-enhanced computed tomography (CECT) scan showed a large multiloculated abdominopelvic cystic mass in the right ovary with thick enhancing septae, calcifications, and a suspicious solid component. The right ovary was not visualized separately. The serum CA-125 level was markedly elevated at >1265 U/mL (Reference range: 0-35 U/mL). Clinical diagnosis of ovarian malignancy was made, and the decision of total abdominal hysterectomy with bilateral salpingo-oophorectomy (TAH-BSO) was made.

**Figure 1:**
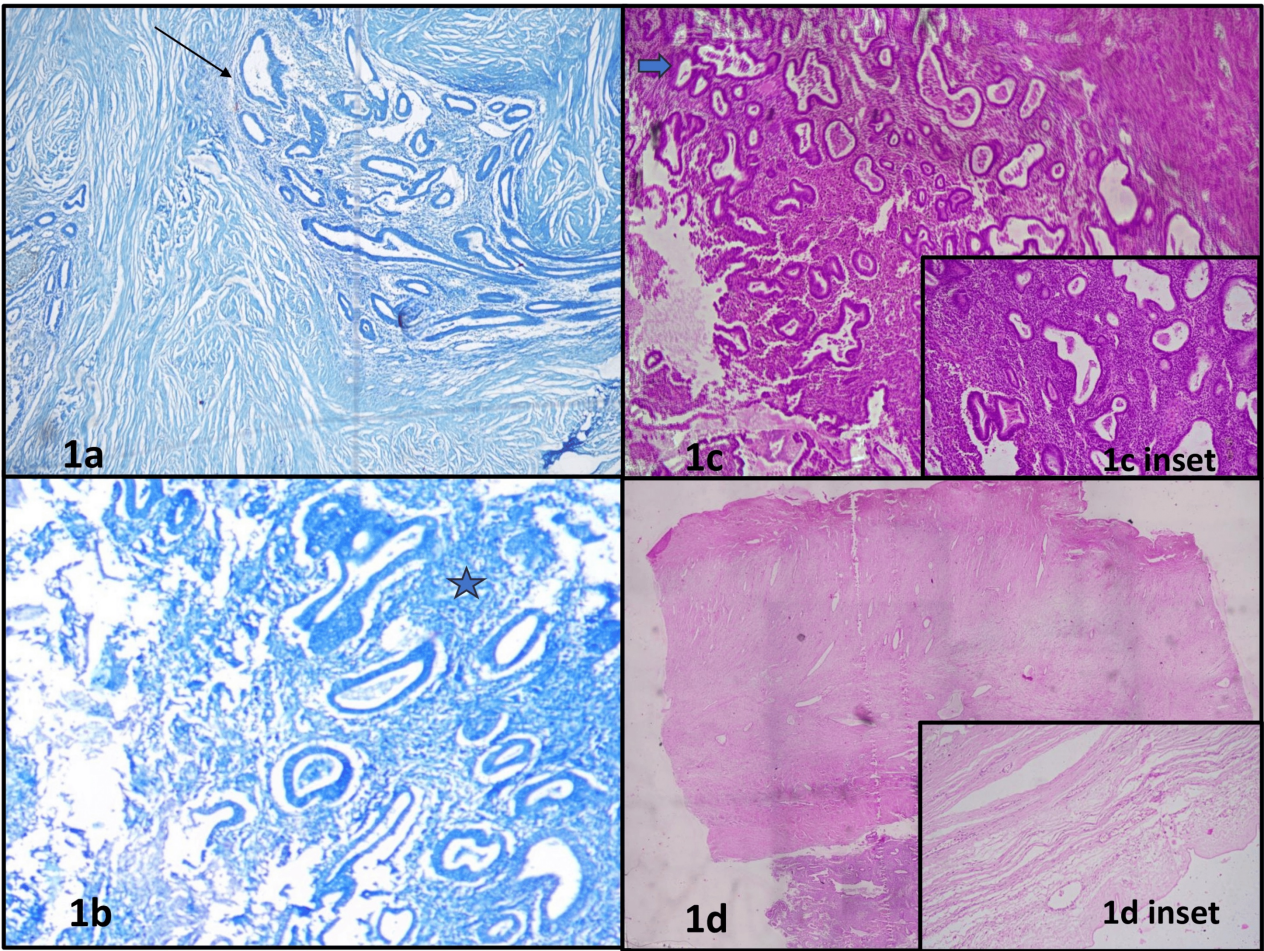
Frozen and light microscopy showing endometrial glands and stroma Figure 1a – Frozen section showing round to tubular endometrial glands (Black thin arrow) and stroma. Toluidine Blue 100X Figure 1b – Frozen section showing endometrial glands and endometrial stroma (Dark blue star). Toluidine Blue 200X Figure 1c – Section showing endometrial glands with simple hyperplasia without atypia (thick green arrow) and proliferation of endometrial stromal cells. H&E 100X Figure 1c inset – Endometrial glands lined by pseudostratified columnar epithelium dispersed in stroma comprising endometrial stromal cells. H&E 200X Figure 1d – Scanner view showing endometrial glands and stroma with parametrium. H&E 40X Figure 1d inset – Section showing ovarian stroma with fibrocollagenous ovarian cyst wall. H&E 100X

The patient underwent TAH-BSO. The surgical specimen was submitted to the pathology department. Intraoperative frozen section stained with toluidine blue showed endometrial glands and stroma depicting features of infiltrative endometriosis, with no evidence of malignancy (Figure 1a, 1b), contrary to the clinical diagnosis.

Gross examination showed the uterus with cervix and bilateral adnexa measuring 17 × 14 × 6 cm, and a right ovarian mass measuring 13 × 10 × 6 cm. Cut sections of the right ovarian mass revealed multiloculated cysts with wall thickness ranging from 0.2 to 1.5 cm, with focal papillary excrescences.

**Figure 2:**
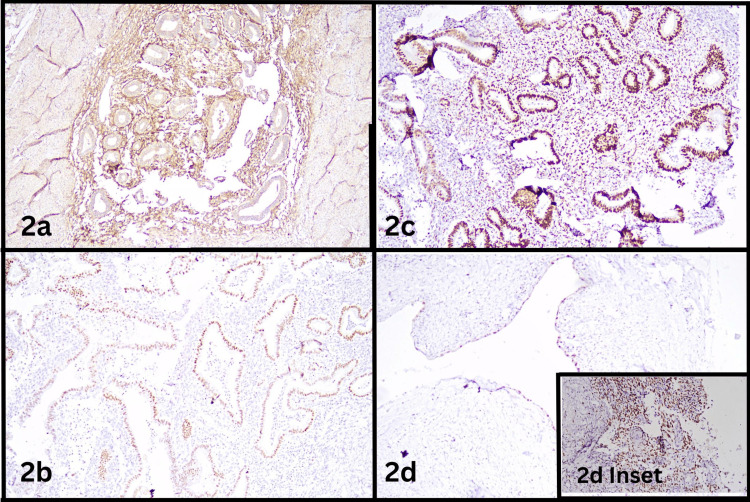
Immunohistochemistry highlighting endometrial glands, endometrial stroma and ovarian stroma. Figure 2a - Endometrial stromal cells showing positivity for CD10 by immunohistochemistry. 200X Figure 2b - Endometrial glands showing positivity for PAX8 by immunohistochemistry. 200X Figure 2c - Endometrial glands and stroma showing positivity for estrogen receptor (ER) by immunohistochemistry. 200X Figure 2d - Ovarian cyst lined by flattened epithelium showing positivity of PAX8 by immunohistochemistry. 200X Figure 2d inset - Ovarian stroma showing positivity for CD10 by immunohistochemistry. 200X

Histopathological examination of the cystic areas of the ovary demonstrated endometrial glands lined by pseudostratified columnar epithelium dispersed in stroma comprising endometrial stromal cells (Figure 1c) with focal simple hyperplasia without atypia (Figure 1c inset) involving the right parametrium (Figure 1d). Adjacent areas also showed ovarian stroma and fibrocollagenous cyst wall (Figure 1d inset). Further immunohistochemistry was performed highlighting endometrial stroma by cluster of differentiation-10 (CD10) (Figure 2a), endometrial glands by Paired Box Gene 8 (PAX8) (Figure 2b). Both endometrial glands and stroma showed positivity for Estrogen Receptor (Figure 2c). Ovarian cyst lined by flattened epithelium showed positivity for PAX8 (Figure 2d). Ovarian stroma showed positivity for CD10 (Figure 2d inset). Additional findings included chronic cervicitis, proliferative endometrium, and an unremarkable myometrium. The bilateral fallopian tubes and left ovary appeared unremarkable, as did part of the right ovary, left parametrium, and bilateral paracervical tissues. Sixteen lymph nodes were identified and showed reactive lymphoid hyperplasia.

A final diagnosis of infiltrative endometriosis with focal simple hyperplasia without atypia was made.

## Discussion

Endometriosis typically affects women of reproductive age. In peri- and postmenopausal women, the decline in estrogen levels reduces endometrial proliferation, making endometrioma an uncommon diagnosis in this age group [5]. Sharadha et al. reported the mean age of patients with benign ovarian tumors to be 39 years, compared to 41 years for malignant ovarian tumors [6]. Therefore, the presentation of an ovarian mass in our 40-year-old patient initially raised a strong suspicion of ovarian malignancy.

Endometriosis is observed in up to 30% of women with infertility and in up to 45% of those with chronic pelvic pain [7]. It is also associated with pain during menstruation (dysmenorrhea), sexual intercourse (dyspareunia), or urination (dysuria). In the present case, the patient, although fertile (P2L2), presented only with pelvic pain and dysmenorrhea, without other typical symptoms of endometriosis.

Ultrasound is typically the first-line, non-invasive diagnostic modality for assessing ovarian masses. A “ground-glass” appearance on ultrasound is suggestive of endometriosis. However, in women over 45 years of age, atypical features such as multilocularity and the presence of solid components are more commonly encountered [8]. In our 40-year-old patient, such atypical features were observed, suggesting a possible ovarian neoplasm. Further evaluation with contrast-enhanced computed tomography (CECT) also revealed a multiloculated solid-cystic mass, reinforcing the suspicion of malignancy.

A serum CA-125 level greater than 35 IU/mL is typically used as a threshold for diagnosing ovarian malignancy. Elevated CA-125 levels can also be seen in endometriosis, with values increasing in correlation with disease severity. According to the American Society for Reproductive Medicine, mean CA-125 levels in stages I to IV of endometriosis are approximately 18.8 ± 0.9 IU/mL, 40.3 ± 2.8 IU/mL, 77.1 ± 3.5 IU/mL, and 182.4 ± 14.0 IU/mL, respectively [9]. Although rare, some cases have shown CA-125 levels far exceeding these averages. In our case, the CA-125 level was markedly elevated at 1265 IU/mL - strongly suggestive of malignancy.

Frozen section analysis is known to be highly accurate in differentiating between benign and malignant ovarian tumors [10]. In this case, however, frozen section unexpectedly revealed infiltrative endometriosis rather than a neoplastic lesion.

Histopathological examination remains the gold standard for diagnosing endometriosis, with the identification of endometrial glands and stroma being confirmatory. Ovarian endometriosis with atypical features carries an increased risk of malignant transformation. Additionally, studies have documented the presence of hyperplasia and atypia within endometriotic lesions [11]. In our case, histopathology revealed simple hyperplasia of the endometrium without atypia. All examined lymph nodes were free of disease, confirming the absence of malignancy.

## Conclusions

This case report emphasizes the challenges faced in diagnosing infiltrative endometriosis, particularly when it presents atypically. A large ovarian mass in a perimenopausal woman, accompanied by imaging findings and markedly elevated CA-125 levels, can closely mimic an ovarian neoplasm. This highlights the importance of considering endometriosis as a differential diagnosis in such scenarios. Furthermore, the case emphasizes the pivotal role of intraoperative frozen section in avoiding radical surgery and definitive histopathological examination in establishing an accurate diagnosis and guiding appropriate clinical management.
